# Mapping wind erosion hazard with regression-based machine learning algorithms

**DOI:** 10.1038/s41598-020-77567-0

**Published:** 2020-11-24

**Authors:** Hamid Gholami, Aliakbar Mohammadifar, Dieu Tien Bui, Adrian L. Collins

**Affiliations:** 1 Department of Natural Resources Engineering, University of Hormozgan, Bandar-Abbas, Hormozgan, Iran; 2 Institute of Research and Development, Duy Tan University, Da Nang, 550000 Vietnam; 3 GIS Group, Department of Business and IT, University of South-Eastern Norway, 3800 Bø i Telemark, Norway; 4 Sustainable Agriculture Sciences, Rothamsted/Research, North Wyke, Okehampton, EX20 2SB Devon UK

**Keywords:** Environmental sciences, Natural hazards

## Abstract

Land susceptibility to wind erosion hazard in Isfahan province, Iran, was mapped by testing 16 advanced regression-based machine learning methods: Robust linear regression (RLR), Cforest, Non-convex penalized quantile regression (NCPQR), Neural network with feature extraction (NNFE), Monotone multi-layer perception neural network (MMLPNN), Ridge regression (RR), Boosting generalized linear model (BGLM), Negative binomial generalized linear model (NBGLM), Boosting generalized additive model (BGAM), Spline generalized additive model (SGAM), Spike and slab regression (SSR), Stochastic gradient boosting (SGB), support vector machine (SVM), Relevance vector machine (RVM) and the Cubist and Adaptive network-based fuzzy inference system (ANFIS). Thirteen factors controlling wind erosion were mapped, and multicollinearity among these factors was quantified using the tolerance coefficient (TC) and variance inflation factor (VIF). Model performance was assessed by RMSE, MAE, MBE, and a Taylor diagram using both training and validation datasets. The result showed that five models (MMLPNN, SGAM, Cforest, BGAM and SGB) are capable of delivering a high prediction accuracy for land susceptibility to wind erosion hazard. DEM, precipitation, and vegetation (NDVI) are the most critical factors controlling wind erosion in the study area. Overall, regression-based machine learning models are efficient techniques for mapping land susceptibility to wind erosion hazards.

## Introduction

Wind erosion, as an environmental problem, has many adverse effects on the economics of societies and the health of terrestrial and marine ecosystems^1–3^. Therefore, predicting land susceptibility to wind erosion hazards such as dust emissions from land surfaces is essential for mitigating its effects. Literature review shows that different tools and techniques have been proposed for investigating different aspects of wind erosion and its consequences, uniquely identifying regions prone to generating sediments for wind erosion, including remote sensing, data mining, and sediment fingerprinting^4–7^. However, these techniques require intensive field sampling with expensive laboratory analyses^8^, and as a result, they are not efficient for large spatial domains.

Recently, together with developments of geospatial technology and computer sciences, machine learning (ML) has received considerable attention with many successful applications in the spatial mapping of different environmental hazards such as land subsidence, gully erosion, landslides, and dust provenance, as well as mapping of soil properties (microbial dynamics, moisture, shear strength, soil taxa, bulk density, total nitrogen, organic carbon). However, to the best of our knowledge, exploration of the utility of advanced ML techniques in predicting land susceptibility to wind erosion has not been undertaken.

Typical ML models applied to date in different areas of environmental research include decision tree and linear equation models, the particle swarm optimization-adaptive network-based fuzzy inference system (PANFIS), genetic algorithms, support vector regression (SVR), artificial neural networks (ANN), hybrid models, random forest (RF), Wang and Mendel's (WM), partial least square regression (PLSR), principal component regression (PCR), Cubist, Bayesian additive regression trees (BART), radial basis function (RBF), extreme gradient boosting (XGBoost) and regression tree analysis^8–15^. Since, to date, a comprehensive study applying regression-based ML models to mapping wind erosion hazard has not been investigated, there remains a need for such work since wind erosion hazards are a major socio-economic challenge for some parts of the world. Accordingly, this work aimed to address this gap in the existing literature by providing a comprehensive assessing of the prediction performance of 16 regression-based ML models (robust linear regression (RLR), Cforest, non-convex penalized quantile regression (NCPQR), neural network with feature extraction (NNFE), monotone multi-layer perception neural network (MMLPNN), ridge regression (RR), boosting generalized linear model (BGLM), negative binomial generalized linear model (NBGLM), boosting generalized additive model (BGAM), spline generalized additive model (SGAM), spike and slab regression (SSR), stochastic gradient boosting (SGB), support vector machine (SVM), relevance vector machine (RVM), Cubist and adaptive network-based fuzzy inference system (ANFIS)) for mapping land susceptibility to the wind erosion hazard in the Isfahan province, central Iran. Using this case study, we provide more generic recommendations.

## Results

### Multicollinearity test

Table1 shows the values of the tolerance coefficient (TC) and the variance inflation factor (VIF) for the controlling factors for wind erosion. VIF > 10 and TC < 0.1 indicate multicollinearity among the effective factors. Based on our results, the lowest TC value was obtained for electrical conductivity (EC), while the highest VIF value (5.93) value was calculated for bulk density. The results indicated the absence of any multicollinearity between the 13 factors controlling wind erosion in the study area.

**Table 1:** Values of the TC and VIF for examining multicollinearity among the effective factors for wind erosion using the training dataset.

Effective factors	Collinearity test	
	TC	VIF
AWC	0.199	4.972
Bulk density	0.169	5.93
Calcium carbonate percentage	0.154	5.291
DEM	0.183	5.46
EC	0.109	5.61
ESP	0.118	5.546
Land use	0.855	1.169
Geology	0.637	1.57
Precipitation	0.315	3.177
Organic carbon content	0.159	5.255
NDVI	0.597	1.674
Soil texture	0.203	4.932
Wind speed (m/s)	0.656	1.523

### Relative importance of the factors affecting wind erosion

**Figure 1:**
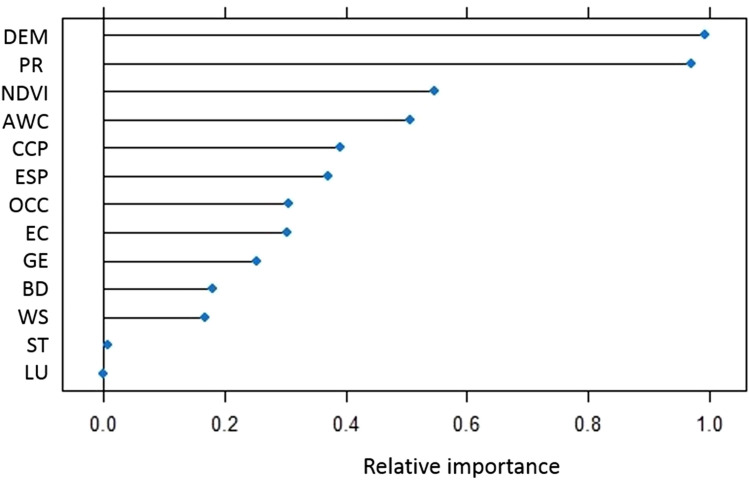
The relative importance of the effective factors for wind erosion estimated by MMLPNN. DEM, PR, NDVI, AWC, CCP, ESP, OCC, EC, GE, BD, WS, ST, and LU indicate digital elevation model, precipitation, normalized difference vegetation index, available water content, calcium carbonate content, exchangeable sodium percentage, organic carbon content, electrical conductivity, geology, bulk density, wind speed, soil texture, and land use, respectively.

The model with the highest performance (MMLPNN) was applied to quantify the relative importance of the effective factors for wind erosion. Based on Fig. 1, three factors, DEM (with relative importance 0.95), precipitation (with relative importance 0.8), and NDVI (with relative importance 0.54), were recognized as the most important factors controlling wind erosion in the study area. Wind erosion has been shown to be affected by many factors such as wind, precipitation, temperature, soil properties (texture, composition, and aggregation), topography, aerodynamic roughness, vegetation, and land use practice^16^.

## Discussion

### Maps of wind erosion hazard

**Figure 2:**
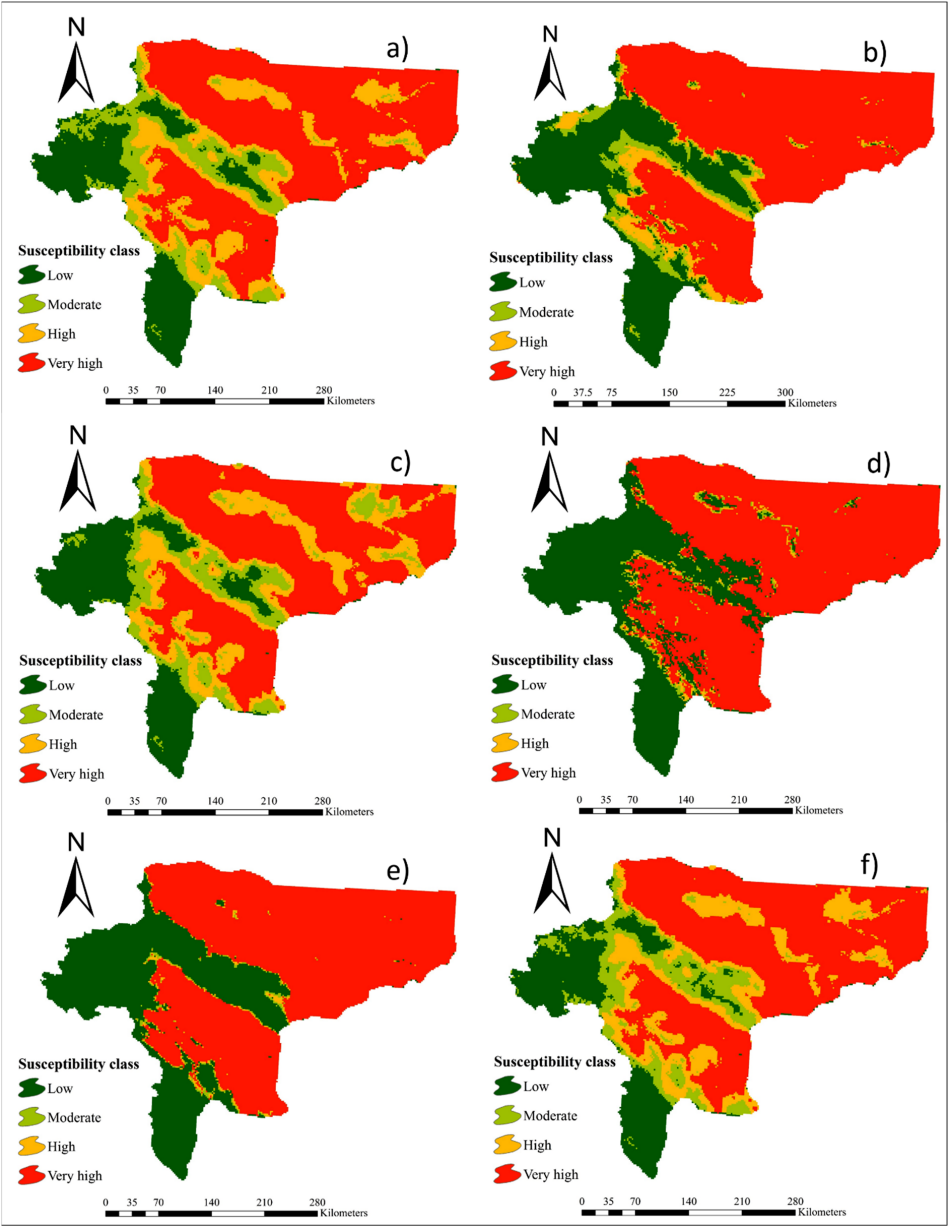
Maps of wind erosion hazard generated by: **(a)** RLR, **(b)** Cforest, **(c)** NCPQR, **(d)** NNFE, **(e)** MMLPNN, and **(f)** RR. The values for pixels was estimated by R software (https://CRAN.R-project.org/doc/FAQ/R-FAQ.html) and then, values of pixels were mapped by ArcGIS 10.4.1 (https://www.esri.com/en-us/about/about-esri/overview).

**Figure 3:**
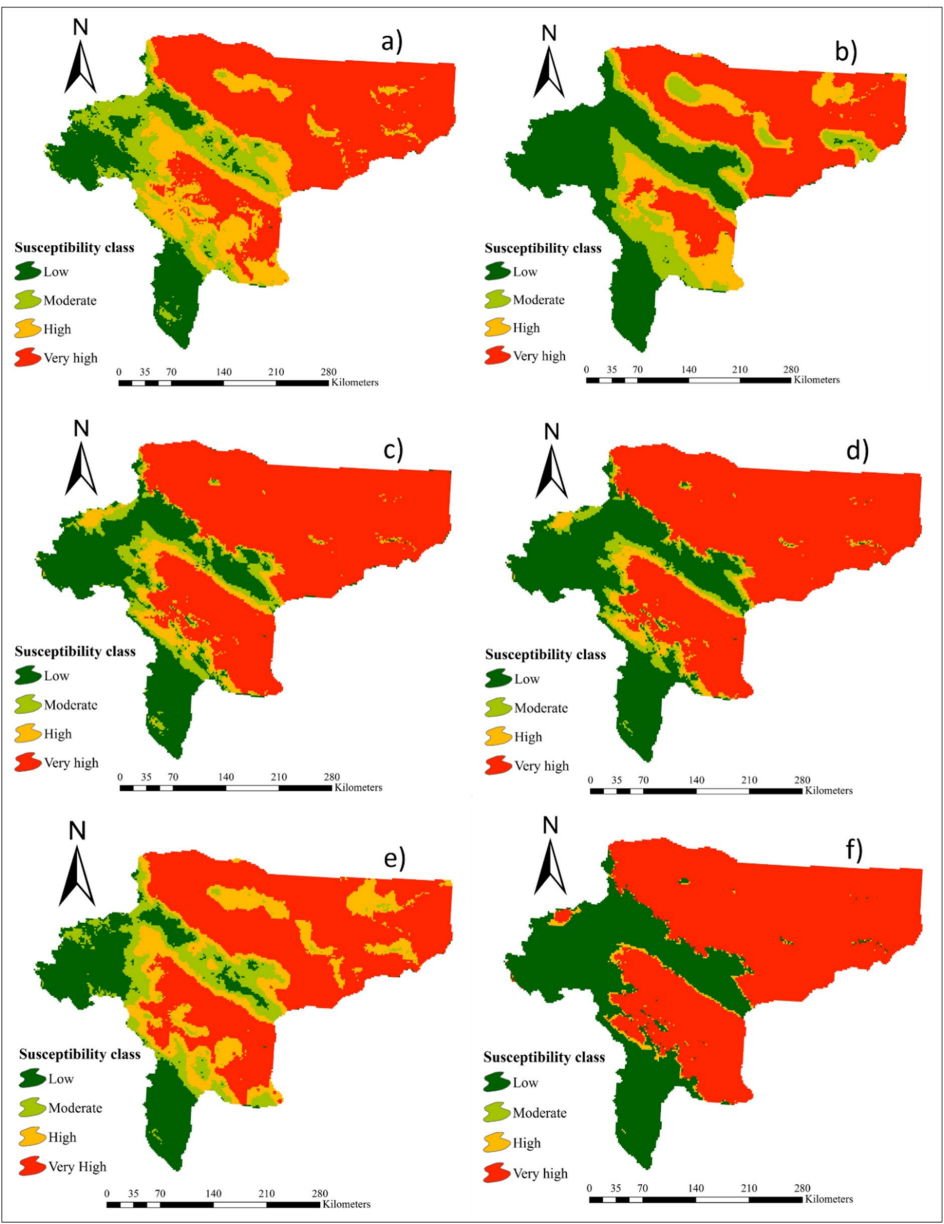
Maps of wind erosion hazard generated by: **(a)** BGLM, **(b)** NBGLM, **(c)** BGAM, **(d)** SGAM, **(e)** SSR, and **(f)** SGB. The values for pixels was estimated by R software (https://CRAN.R-project.org/doc/FAQ/R-FAQ.html) and then, values of pixels were mapped by ArcGIS 10.4.1 (https://www.esri.com/en-us/about/about-esri/overview).

**Figure 4:**
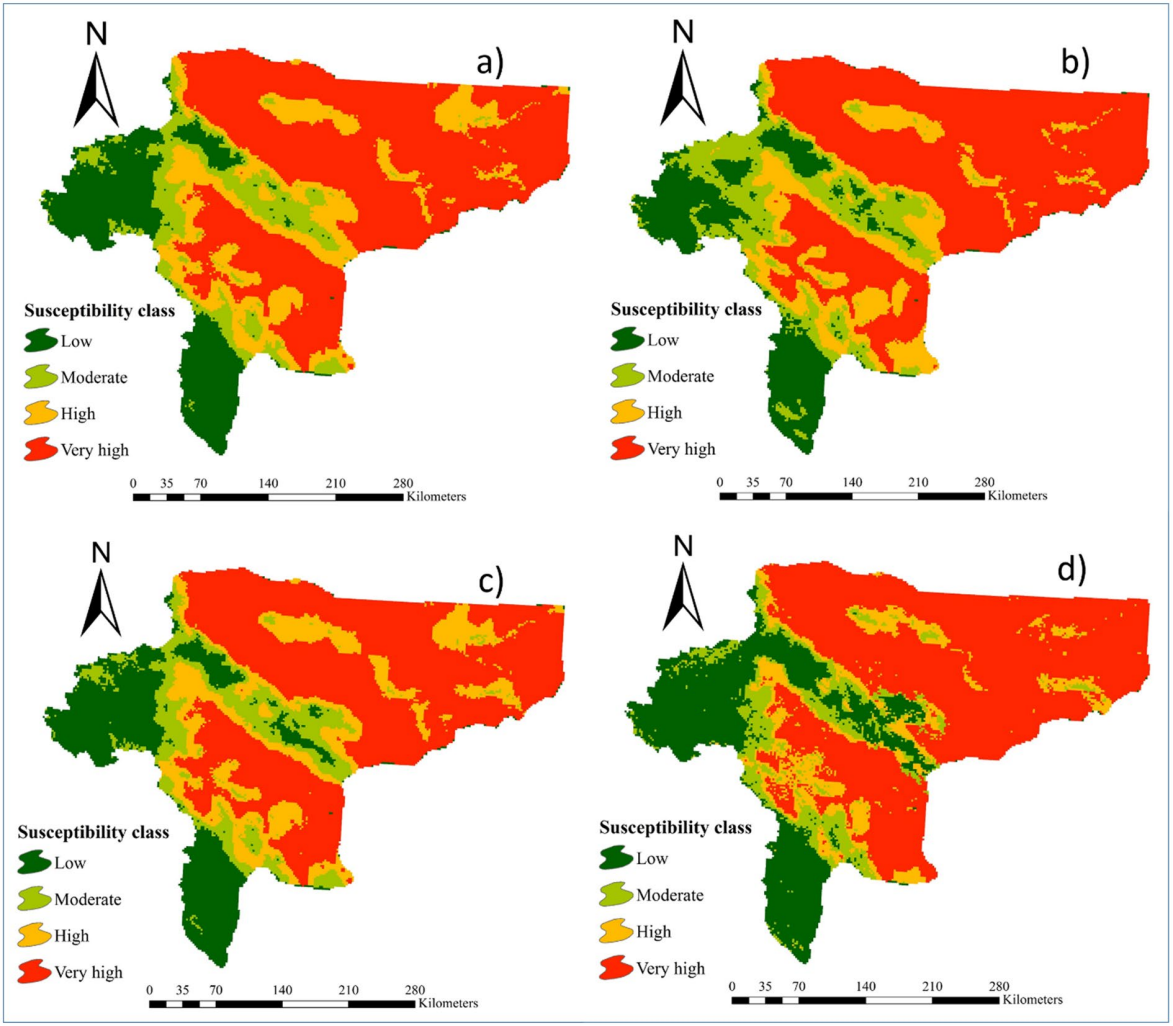
Maps of wind erosion hazard generated by: **(a)** SVM, **(b)** RVM, **(c)** Cubist and **(d) **ANFIS. The values for pixels was estimated by R software (https://CRAN.R-project.org/doc/FAQ/R-FAQ.html) and then, values of pixels were mapped by ArcGIS 10.4.1 (https://www.esri.com/en-us/about/about-esri/overview).

The wind erosion hazard maps generated by 16 individual ML models are presented in Figs.2, 3, and 4. Table 2 indicates the area (percentage and km^2^) of the four land susceptibility classes (low, moderate, high, and very high) for wind erosion hazard estimated by the 16 ML models. Based on the results of all 16 models, areas of land susceptibility to the low susceptibility class ranged between 15.5% (RVM and BGLM models) and 32.8% (MMLPNN model). The minimum and maximum areas of moderate land susceptibility to wind erosion were estimated by the SGB (0.6%) and SSR (15.7%) models, respectively. The area of land categorized into the high susceptibility class ranged from 1.2% (MMLPNN model) to 20.2% (NCPQR model). Corresponding areas assigned to the very high class of land susceptibility to wind erosion hazard ranged from 41% (NBGLM model) to 65.2% (SGB).

**Table 2:** Land susceptibility classes to wind erosion hazard calculated by 16 individual ML models.

Model	Susceptibility class							
	Low		Moderate		High		Very high	
	Area (km^2^)	Area (%)	Area (km^2^)	Area (%)	Area (km^2^)	Area (%)	Area (km^2^)	Area (%)
RLR	18,870	17.7	13,168	12.3	18,742	17.5	55,965	52.5
Cforest	27,759	26	7101	6.4	7084	6.6	64,779	61
NCPQR	21,076	19.7	12,963	12.1	21,554	20.2	51,174	48
NNFE	33,748	31.6	1717	1.6	1894	1.8	69,429	65
MMLPNN	35,007	32.8	1195	1.1	1296	1.2	69,295	64.9
RR	18,857	17.7	12,945	12.1	19,470	18.2	55,454	52
BGLM	16,356	15.4	15,126	14.2	20,484	19.2	54,474	51.2
NBGLM	32,702	30.5	13,433	12.6	16,992	15.9	43,646	41
BGAM	24,767	23.2	8295	7.8	7545	7	66,147	62
SGAM	29,148	27.4	5957	5.6	5752	5.4	65,627	61.6
SSR	18,894	17.7	16,719	15.7	23,510	22	47,562	44.6
SGB	34,541	32	60	0.6	2310	2.2	69,844	65.2
SVM	18,214	17	11,382	10.7	20,374	19	56,783	53.3
RVM	16,364	15.4	15,767	14.8	18,523	17.3	56,098	52.5
Cubist	19,326	18.2	12,543	11.7	19,797	18.5	55,077	51.6
ANFIS	24,022	22.6	10,391	9.8	12,873	12	59,167	55.6

### Model performance assessment

**Figure 5:**
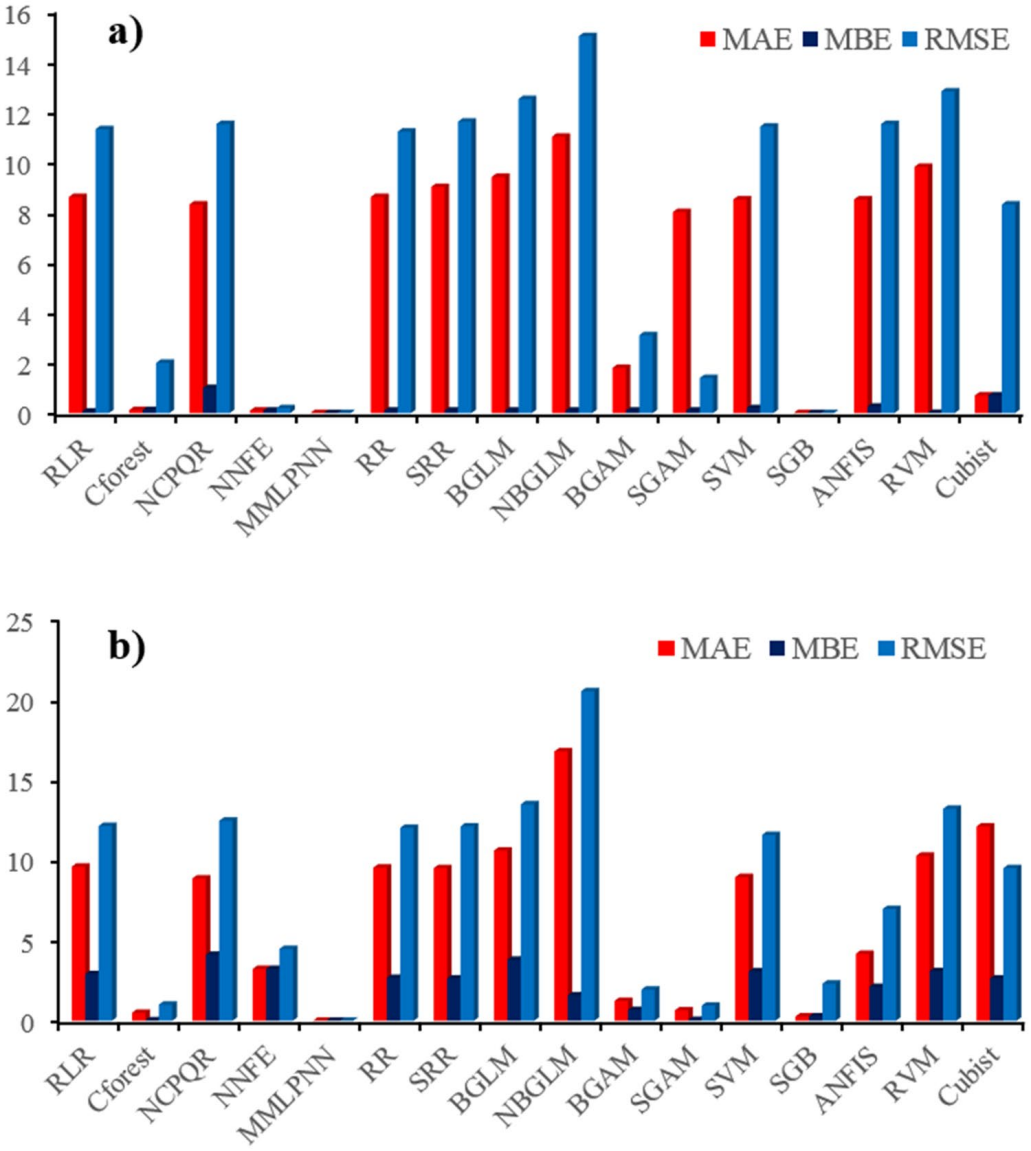
The values of the statistical indicators were used to evaluate model performance; **(a)** training dataset and **(b)** evaluation dataset.

**Figure 6:**
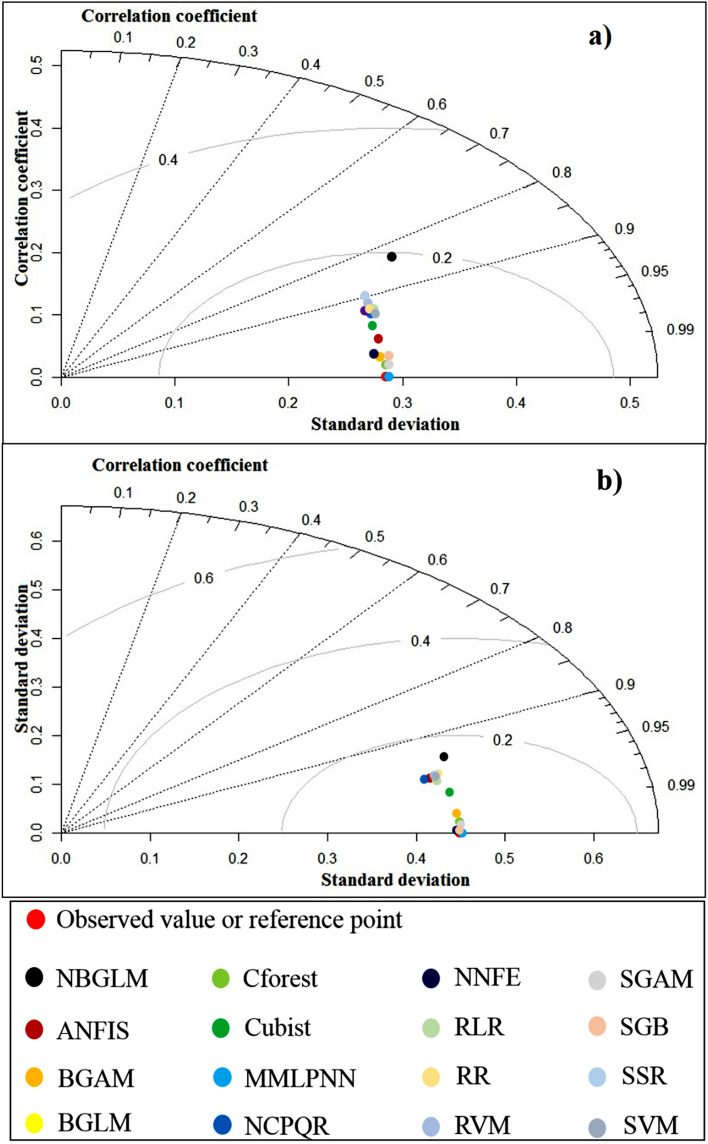
Taylor diagrams for assessing the performance of the models in this research; **(a)** training dataset, and **(b)** evaluation dataset.

Model performance for mapping wind erosion hazard was assessed by three indices (MAE, MBE, and RMSE; (Fig. 5)). Additionally, a Taylor diagram for both the training and evaluation datasets were constructed (Fig. 6). MMLPNN was selected as the most accurate model for mapping wind erosion hazard, while according to the RMSE and MAE, NBGLM was the weakest predictive model, and NCPQR was recognized as the overall worst model.

Based on all three statistical indicators of model performance and the Taylor diagram for the evaluation dataset, five models (MMLPNN, SGAM, Cforest, BGAM, and SGB) returned low errors. SSR and NBGLM had the lowest accuracies among the 16 models. Based on the Taylor diagram drawn for the training dataset, five models (MMLPNN, Cforest, SGAM, SGB and NNFE) were identified as the most accurate predictive ML models for mapping wind erosion hazard in the study area, whereas NBGLM and RVM were the weakest predictive models.

Overall, MMLPNN, SGAM, Cforest, BGAM, and SGB were identified as the most accurate models for predicting land susceptibility to wind erosion. Based on MMLPNN (Fig. 2e), the four susceptibility classes covered 32.8%, 1.1%, 1.2% and 64.9% of the total area of Isfahan province, respectively. The land susceptibility map to wind erosion hazard generated using SGAM shows the high and very high susceptibility classes covered 5.4% and 61.5% of the total area, respectively, whereas the low and moderate susceptibility classes occupied 27.4% and 5.6%, respectively (Fig. 3d). According to Cforest (Fig. 2b), 26%, 6.4%, 6.6%, and 61% of the total area belonged to the low, moderate, high and very high susceptibility classes, respectively. Using BGAM (Fig. 3c), the very high susceptibility class covered 62% of the study area, whereas the low, moderate, and high classes occupied 23.2%, 7.8% and 7% of the total area, respectively. Finally, in the case of the SGB model (Fig. 3f), the results classified 32%, 0.6%, 2.2% and 65.2% of the study area as low, moderate, high, and very high susceptibility, respectively.

The map of wind erosion hazard produced by MMLPNN is the most accurate. Overall, multi-layer perception networks (MLPS) as universal estimators are well-known techniques for system identification. The monotonicity of MMLPNN does not depend on the quality of the training data because it is guaranteed by its structure^17^. GAM with spline function (SGAM) was one of the 5 most accurate models for wind erosion hazard mapping. The spline functions allow the flexible representation of non-linear marginal relationships of the explanatory and response variables without the necessity to define a specific function^18^. Cforest, as a random forest (RF) model, uses conditional inference trees for prediction^19^. Several studies confirm the performance of RF as a suitable model for spatial predictions of environmental hazards. For example^20^, reported that the RF model is the best model for digital mapping of soil carbon fractions.

Some studies^21^ have also argued that RF has the highest predictive capability for modelling landslide susceptibility in comparison with other ML models. Some previous studies^22^ have also reported that in comparison with other methods, RF has better performance in estimating PM_2.5_ monthly concentration. In this study, we applied the boosting with generalized additive model (BGAM), and based on the indicators for examining model performance, this model exhibited satisfactory performance and was selected as one of the five most accurate models for mapping wind erosion hazard. Boosting is a technique for improving prediction rules, and it can be applied to classification and regression methods to increase the accuracy of the predictions^23^. SGB is related to both boosting and bagging^24,25^. Previous research^26^ has reported that SGB provides stable predictions for tree species presence.

## Conclusions

This research assessed the performance of 16 individual regression-based ML algorithms for mapping land susceptibility to wind erosion hazard in an arid region in central Iran. In all, 13 effective factors for wind erosion were considered and regions with active wind erosion were mapped using a "wind erosion inventory map". Based on three statistical indicators and a Taylor diagram, the MMLPNN model was the most accurate model. We conclude that MMLPNN is powerful tool for mapping wind erosion hazard in arid and semi-arid region ecosystems worldwide. We recommend that future work should focus on testing and comparing the performance of regression-based and classification-based ML models for the mapping and spatial modelling of wind erosion and dust sources to ensure that robust evidence is provided to support management decisions.

## Material and methods

### Study area

**Figure 7:**
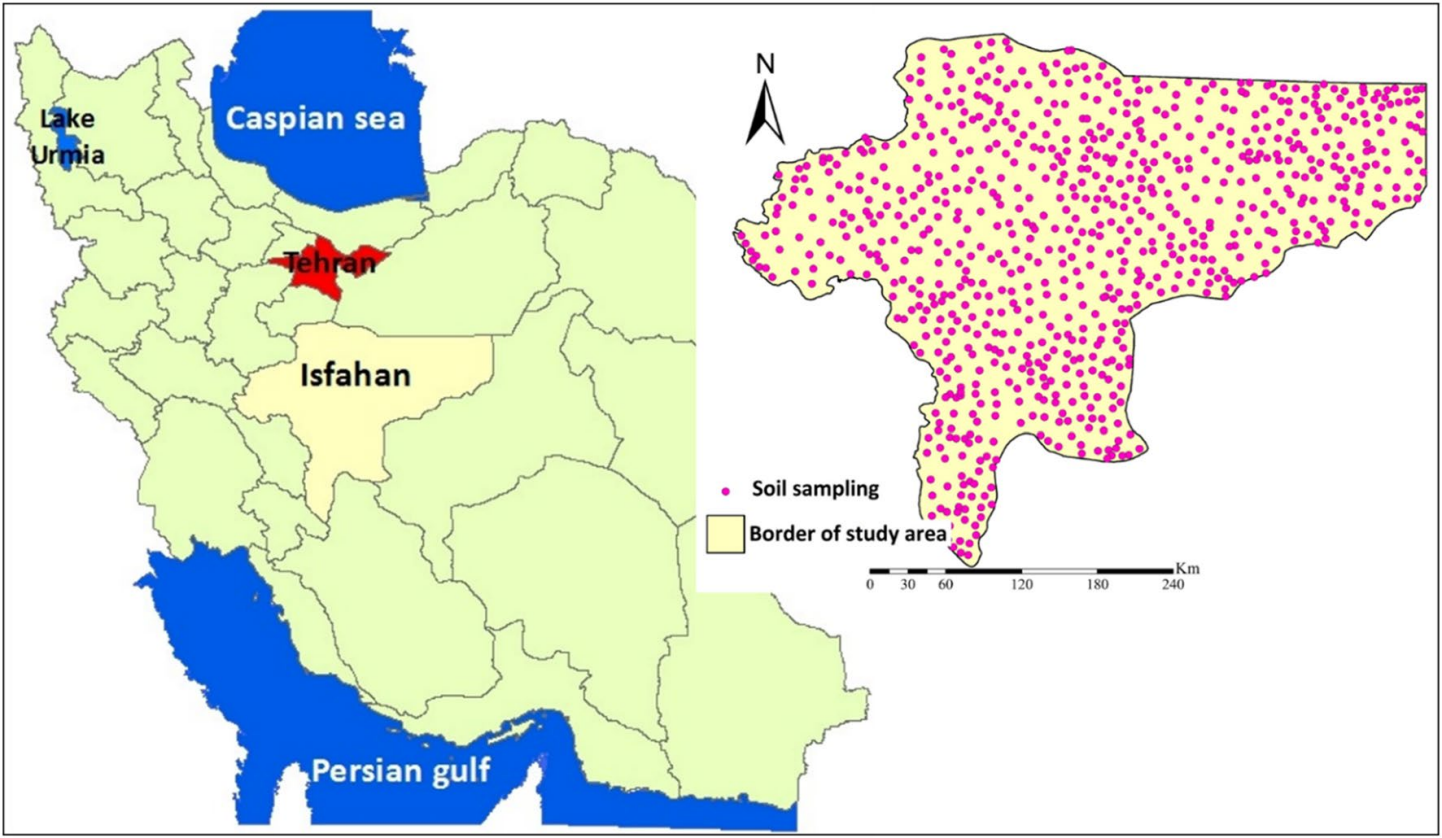
Location of the study area in Iran and sampling sites used for this study. Soil sampling sites were extracted from the world soil map^30^ and then, these sites were mapped in ArcGIS 10.4.1 (https://www.esri.com/en-us/about/about-esri/overview).

Isfahan province (Fig. 7), an arid region, is located in central Iran, between the latitudes 30°45′59.51" to 34°27′13.27" N, and between the longitudes 49°41′53.86" to 55°30′13.67" E. It is experiencing intensive wind erosion on the southeastern side (Segzi plain) and its northern parts. Based on a digital elevation model (DEM), there is high variability in altitude with maximum and minimum elevations ranging between 686 m (in the northern part of the study area and southern parts of Dasht-e-Kavir) to 4398 m (in the vicinity of the Dena Mountain in the southwestern part of the study area). The average annual precipitation ranges between 72 mm (in the eastern part with a corresponding annual mean temperature of 18 °C) and 320 mm (in the western part with an average annual temperature of 13 °C).

### Factors controlling wind erosion

Different environmental and climatic factors are controlling wind erosion phenomena in drylands. Environmental variables affecting wind erosion include soil properties, lithology, land use, vegetation cover, topography, and elevation^1,8,27^. Previous research^28^ introduced a local wind erosion climatic index based on the wind speed and effective precipitation index developed by^29^ for applying in the Chepil wind erosion equation (WEQ).

### Soil characteristics

**Figure 8:**
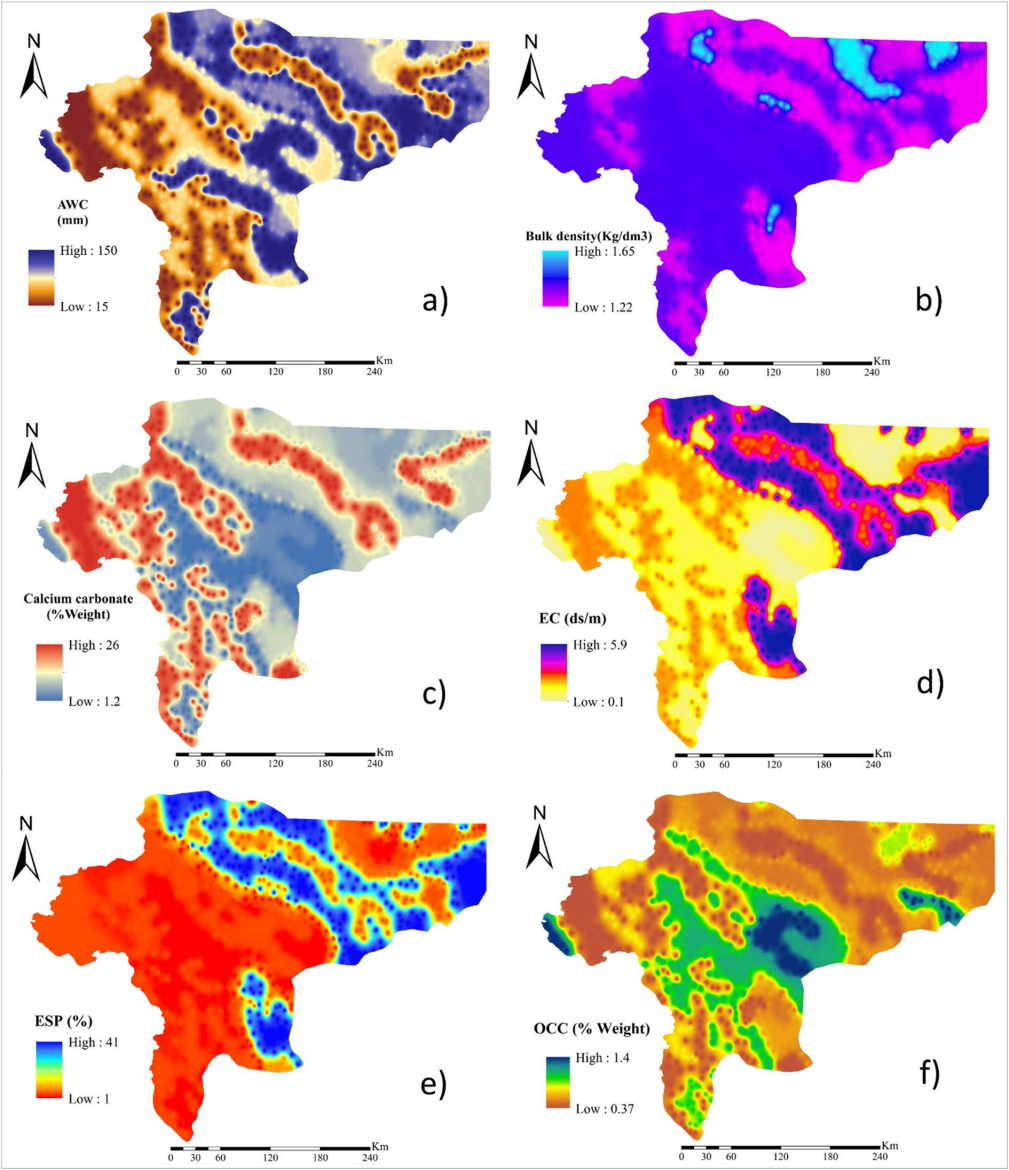
Spatial maps of soil characteristics: **(a)** AWC; **(b)** bulk density; **(c)** calcium carbonate percentage; **(d)** EC; **(e)** ESP, and; **(f)** OCC. All these factors were mapped spatially in ArcGIS 10.4.1 (https://www.esri.com/en-us/about/about-esri/overview).

**Figure 9:**
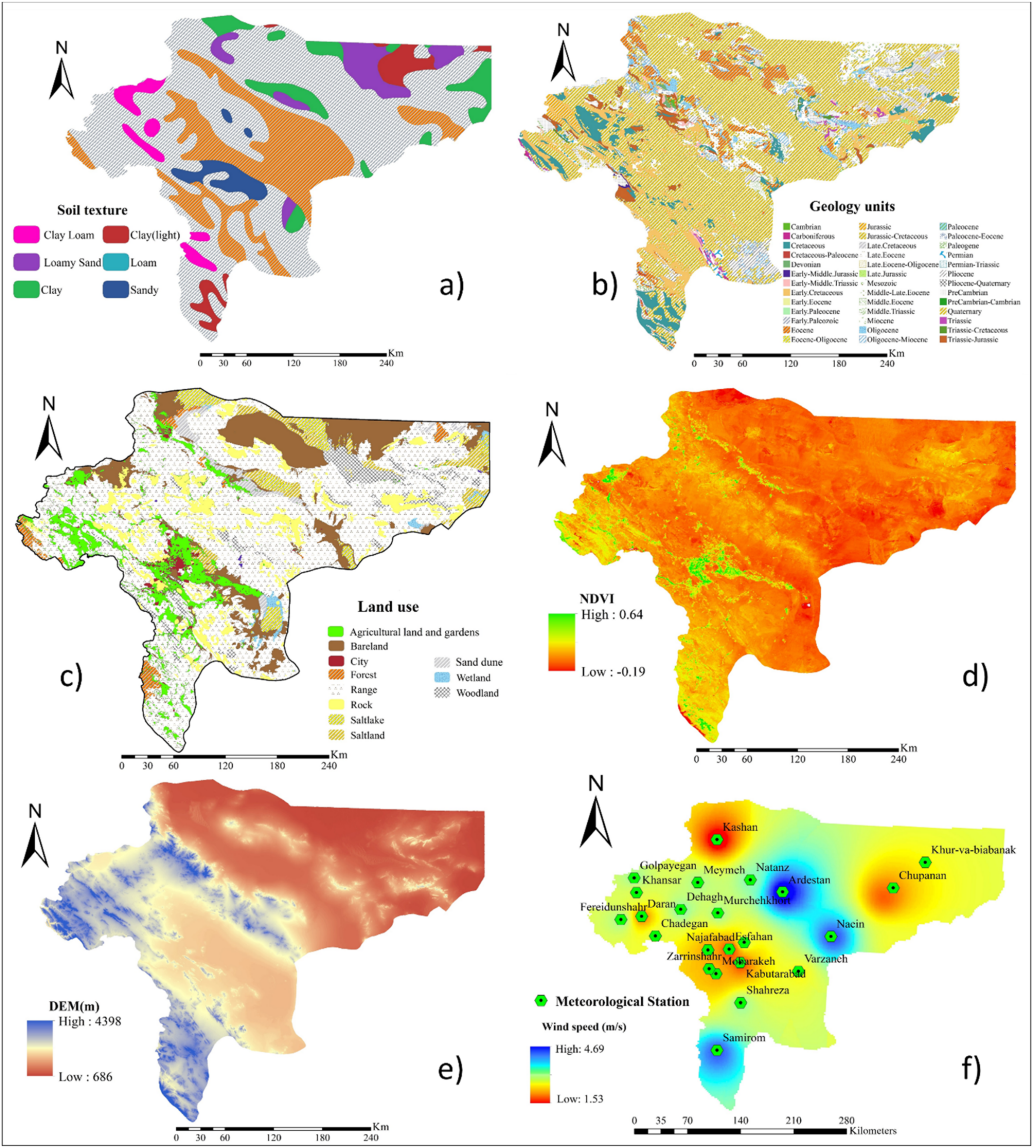
Spatial maps of: **(a)** soil texture; **(b)** geology; **(c)** land use; **(d)** NDVI; **(e)** DEM, and; **(f)** wind speed. All these factors were mapped spatially in ArcGIS 10.4.1 (https://www.esri.com/en-us/about/about-esri/overview).

Seven soil characteristics (e.g., available water content (AWC) (Fig. 8a), bulk density (Fig. 8b), calcium carbonate percentage (Fig. 8c), electrical conductivity (EC) (Fig. 8d), exchangeable sodium percentage (ESP)(Fig. 8e), organic carbon content (OCC)(Fig. 8f) and soil texture (Fig. 9a)) were extracted from the world soil map^30^ and mapped by interpolation in ArcGIS 10.4.1. It should be noted that a total of 803 points (Fig. 7) were used for generating spatial maps.

### Lithology and land use

Lithology (Fig. 9b) and land use (Fig. 9c) were mapped spatially based on the maps produced by the Forests, Rangelands, and Watershed Management Organization of Iran (FRWMOI).

### Vegetation cover

The normalized difference vegetation index (NDVI) (Fig. 9d)^31^ as the most common index used for the spatial mapping of vegetation cover was applied in our study. NDVI is the difference between the red (RED) and near-infrared (NIR) band combination divided by the sum of the red and near-infrared band combination (Eq. 1).

1 $$ {\text{NDVI }} = \, \left( {{\text{NIR}}_{{{\text{b4}}}} {-}{\text{ RED}}_{{{\text{b3}}}} } \right) \, / \, \left( {{\text{NIR}}_{{{\text{b4}}}} + {\text{ RED}}_{{{\text{b3}}}} } \right) $$

### Elevation

A digital elevation model (DEM) (Fig. 9e) for the study area was generated using shuttle radar topography mission (SRTM) images with a 30*30 m resolution^8^.

### Climatic variables

**Figure 10:**
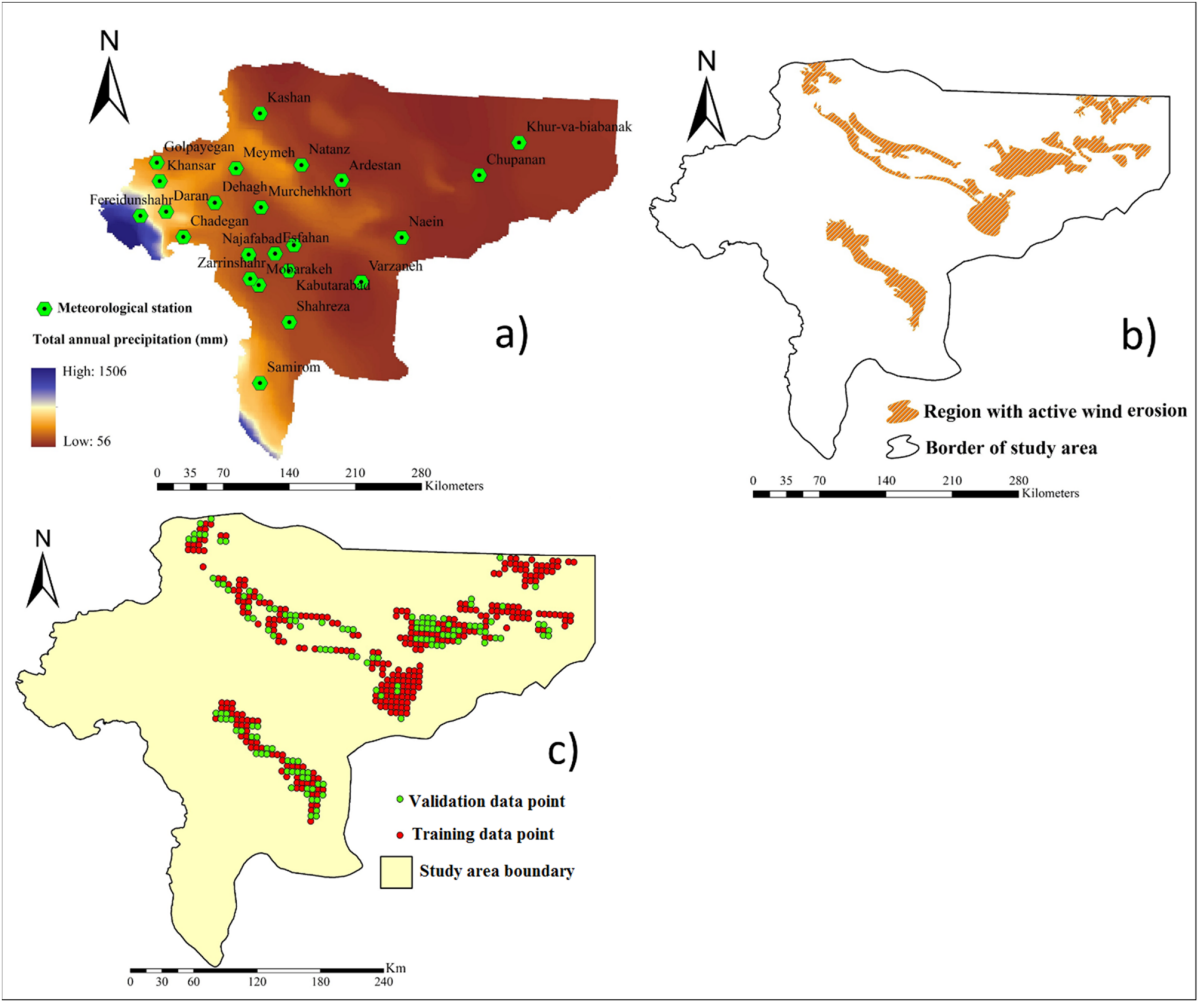
Spatial maps of: **(a)** total annual precipitation; **(b)** locations of the pixels with active wind erosion, and; **(c)** locations of the training and validation data points. All these characteristics were mapped spatially in ArcGIS 10.4.1 (https://www.esri.com/en-us/about/about-esri/overview).

Wind speed (Fig. 9f) and precipitation (Fig. 10a) were used as climatic factors affecting wind erosion. The spatial maps of these variables were generated based on the daily average wind speed and total annual precipitation data from 23 meteorological stations located in the Isfahan province. All spatial maps of factors controlling wind erosion were generated in ArcGIS 10.4.1.

### Inventory map of wind erosion

An inventory map shows regions with active three-stage processes, comprising detachment, transportation, and sedimentation due to wind erosion. An inventory map is needed for predicting land susceptibility to wind erosion hazard. We used a map of regions with active wind erosion produced by the Forest, Rangeland and Watershed Management Organization of Iran (FRWMOI) (Fig. 10b). Based on the inventory map, wind erosion active regions covered ~ 10,961 km^2^ (440 pixels) in the study area. Pixels with active wind erosion were randomly selected for the training (70% or 308 pixels) and validation (30% or 112 pixels) datasets for the ML models (Fig. 10c). Based on field work and FRWMOI, inventory map of wind erosion was generated in ArcGIS 10.4.1.

### Multicollinearity among the factors controlling wind erosion

The tolerance coefficient (TC) (Eq. 2) and variance inflation factor (VIF) (Eq. 3) ^8,15,32^ were applied to examine multicollinearity among the factors for wind erosion in the Isfahan province.

2 $$ {\text{TC }} = { 1 }{-}{\text{ R}}^{{2}} $$

3 $$ {\text{VIF }} = \left[ {\frac{1}{{{\text{TC}}}}} \right] $$

where R^2^ is the regression coefficient. If the TC is < 0.1 and the VIF is > 10, both coefficients signify a multicollinearity problem.

### Background of the ML algorithms used

This section briefly describes the 16 individual regression-based ML algorithms, which were adopted for mapping wind erosion hazard. These algorithms are available in the caret package, in R software.

### Robust linear regression (RLR)

Robust regression is designed to overcome some limitations of traditional parametric and non-parametric methods. Available robust regression methods include M-estimates^33^, R-estimates^34^, least median of squares (LMS) estimates^35^, least trimmed squares (LTS) estimates and S-estimates^36^, generalized S-estimates (GS-estimates)^37^ and MM-estimates^38^. We used a robust linear regression model with M-estimates for predicting land susceptibility to wind erosion.

### Cforest

Random forest (RF), introduced by^39^, is the most popular method for regression and classification in decision tree learning^40^. RF makes a large number of decision trees in the training phase, and then by averaging the output values of the trees, the output of the model is finalized. Cforest is a type of RF commonly applied for prediction purposes^19^.

### Non-convex penalized quantile regression (NCPQR)

Quantile regression (QR) has gained considerable attention in different fields of modelling since the work of^41^. In comparison with mean regression (MR), QR provides an alternative that is more efficient when the error term follows a non-normal heavy-tailed distribution^42^. We used a penalized QR with a non-convex function^42^ for mapping wind erosion hazard.

### Neural networks (NN)

NN can accurately approximate complicated non-linear input/output relationships^43^. The NN structure includes a set of interconnected units or neurons that estimates the non-linear correlations between each variable. The input neurons or predictor variables are connected to a single or multiple layers of hidden neurons, which are then linked to the output neurons^44^. We used a NN with the feature extraction algorithm (NNFE)^45^ and a monotone multi-layer perception neural network (MMLPNN)^46^ for mapping wind erosion hazard. The feature extractors used textural features based on the spatial relationships between pixels^45^.

### Ridge regression with variable selection

Ridge regression *(*RR), which was proposed by^47^, is expressed as follows (Eq. 4):

4 $$L\left(w\right)=\sum_{i=1}^{n}{\left({y}_{i}- {\tilde{y}}_{i}\right)}^{2}=\sum_{i=1}^{n}{({y}_{i}-w. {x}_{i})}^{2}$$

Given a set of n vectors, x_1_, … , x_n_ in R^m^, where m is the number of properties, and the dependent variable y_i_ ∈ *R, i* = *1, …, n,* the objective is to minimize the loss function, i.e., the discrepancy between the real values y_i_ and the predicted values ỹ_i_ = w.x.

We applied a RR model with a kernel function^48^ as follows:

5 $$ {\tilde{y}}=f\left(x\right)=\sum_{i=1}^{n}{\ss }_{i} K \left(x, {x}_{i}\right)$$

where $K \left(x, {x}_{i}\right)$ is the kernel function and β_*i*_ is the weighting.

### Generalized linear models (GLMs)

GLMs have been applied to a wide range of research^49^. GLMs have three components, comprising an observation model, a linear predictor, and an invertible link function^50^. Using boosting with GLMs can improve prediction accuracy^23^. We applied two GLMs; boosting GLM (BGLM) and negative binomial GLM (NBGLM)^51^.

### Generalized additive models (GAMs)

GAMs^52^ can be expressed as follows:

6 $$e\left({\mu }_{i}\right)= {Z}_{i}^{*}.\ss + \sum_{j}{f}_{j} ({x}_{ij})$$

with

7 $${\mu }_{i}=E \left({Y}_{i}\right), and {Y}_{i} \sim EF \left({\mu }_{i}, \varnothing \right),$$

where ${Y}_{i}$ is the *i*th value of the response variable from an exponential distribution family (EF) with a location parameter (${\mu }_{i})$ and a scale parameter ($\varnothing$),${Z}_{i}^{*}$ indicates the *i*th row of a parametric model matrix with the vector β, *f*_*j*_ shows unknown functions and ${x}_{ij}$ indicates the *i*th value of the *j*th variable. $g\left({\mu }_{i}\right)$ is the link function. We applied two GAMs, comprising boosting (BGAM) and spline (SGAM)^18^.

### Spike and slab regression (SSR)

SSR is one of the typical variable selection approaches in regression settings, and this model has been applied widely in challenging problems^53^. SSR was proposed by^54^ and can be expressed as follows^53^:

8 $${y}_{i}={\beta }_{\mathrm{1,0}}{x}_{i,1}+\dots + {\beta }_{p,0}{x}_{i,p}+ {\varepsilon }_{i}, i=1, \dots , n,$$

where (ε_i_)_1≤ i ≤n_ are independent random variables such as E(ε_i_) = 0 and E (${\varepsilon }_{i}^{2}$) = ${\sigma }_{0}^{2}>0.$ Write X for the n × p design matrix corresponding to (1) and ${\beta }_{0}={({\beta }_{\mathrm{0,1}},\dots , {\beta }_{0,P})}^{T}$ for the true regression parameter. The variables ${x}_{i}={({x}_{i,1},\dots , {x}_{i,p})}^{T}$ and the response-vector $y={({y}_{1},\dots , {y}_{n})}^{T}$ are assumed to the standardized such that:$\sum_{i=1}^{n}{x}_{i,k}=0, \sum_{i=1}^{n}{x}_{i,k}^{2}=n, \sum_{i=1}^{n}{y}_{i}=0.$

### Stochastic gradient boosting (SGB)

SGB or gradient boosting machine, proposed by^24^ is a hybrid algorithm that combines both the advantages of bagging and boosting. This model makes additive regression models by the least-squares at each iteration.

### Support and relevance vector machine (SVM and RVM) algorithms

The relevance vector machine (RVM) is a probabilistic sparse kernel model identical in functional form to the support vector machine (SVM). SVM is a very successful approach to supervised learning, and it makes predictions based on the following function^55^:

9 $$y\left(x\right)=\sum_{n=1}^{m}{w}_{n} K \left(x, {x}_{n}\right)+ {w}_{0},$$

where ${w}_{n}$ indicates the model weights and *K* (. , .) is a kernel function. We applied two algorithms, SVM with linear kernel function and RVM with polynomial kernel function.

### Cubist

Cubist, a rule-based regression tree algorithm, is based on the M5 theory^56^. This model involves four main steps as follows: (1) growing a tree by branching data, (2) developing the model, (3) pruning the tree, and (4) smoothing the tree^57^.

### Adaptive network-based fuzzy inference system (ANFIS)

This model has been applied in different sciences. ANFIS works based on the fussy if/then rules^58^:

10 $$ {\text{Rule 1}}:{\text{ if }}\left( {{\text{x is A}}_{{1}} } \right){\text{ and }}\left( {{\text{y is B}}_{{1}} } \right){\text{ then }}\left( {{\text{f}}_{{1}} = {\text{ p}}_{{1}} {\text{x }} + {\text{ q}}_{{1}} {\text{y }} + {\text{ r}}_{{1}} } \right) $$

11 $$ {\text{Rule 2}}:{\text{ if }}\left( {{\text{x is A}}_{{2}} } \right){\text{ and }}\left( {{\text{y is B}}_{{2}} } \right){\text{ then }}\left( {{\text{f}}_{{2}} = {\text{ p}}_{{2}} {\text{x }} + {\text{ q}}_{{2}} {\text{y }} + {\text{ r}}_{{2}} } \right) $$

where x and y are as input parameters for FIS, f as FIS output, A and B are fuzzy sets, and p, q, and r are parameters.

In all 16 models, the predicted values for pixels ranged between 0–1. Therefore, we can divide susceptibility predictions into four classes (low (0–0.25), moderate (0.25–0.50), high (0.50–0.75) and very high (0.75–1)).

### Assessment of model performance

In order to evaluate model performance in predicting land susceptibility to wind erosion hazard in the study area, three statistical methods comprising root mean square error (RMSE), mean absolute error (MAE)^59,60^ and mean bias error (MBE) were used:

12 $$RMSE=\sqrt{\frac{\sum_{i=1}^{m}({v}_{k}-{v}_{p}{)}^{2}}{m}}$$

13 $$MAE= \frac{\sum_{i=1}^{m}\left|{v}_{k}-{v}_{p}\right|}{m}$$

14 $$MBE= \frac{1}{m} \sum_{i=1}^{m}({v}_{k}- {v}_{p})$$

where *m* is number of the observations, ${v}_{k}$ and ${v}_{p}$ indicate the measured and predicted values, respectively. Also, a Taylor diagram was applied as a further test for assessing the performance of individual regression-based ML models^14^.

### Prioritization of the factors controlling wind erosion

Among the 16 ML models tested, a model with the lowest error (RMSE, MAE, and MBE) was applied to quantify the relative importance of the factors controlling wind erosion. In this study, MMLPNN had the lowest error (with RMSE, MAE, and MBE < 0.002%) and was therefore applied for determining the relative importance of the factors for wind erosion.

**Figure 11:**
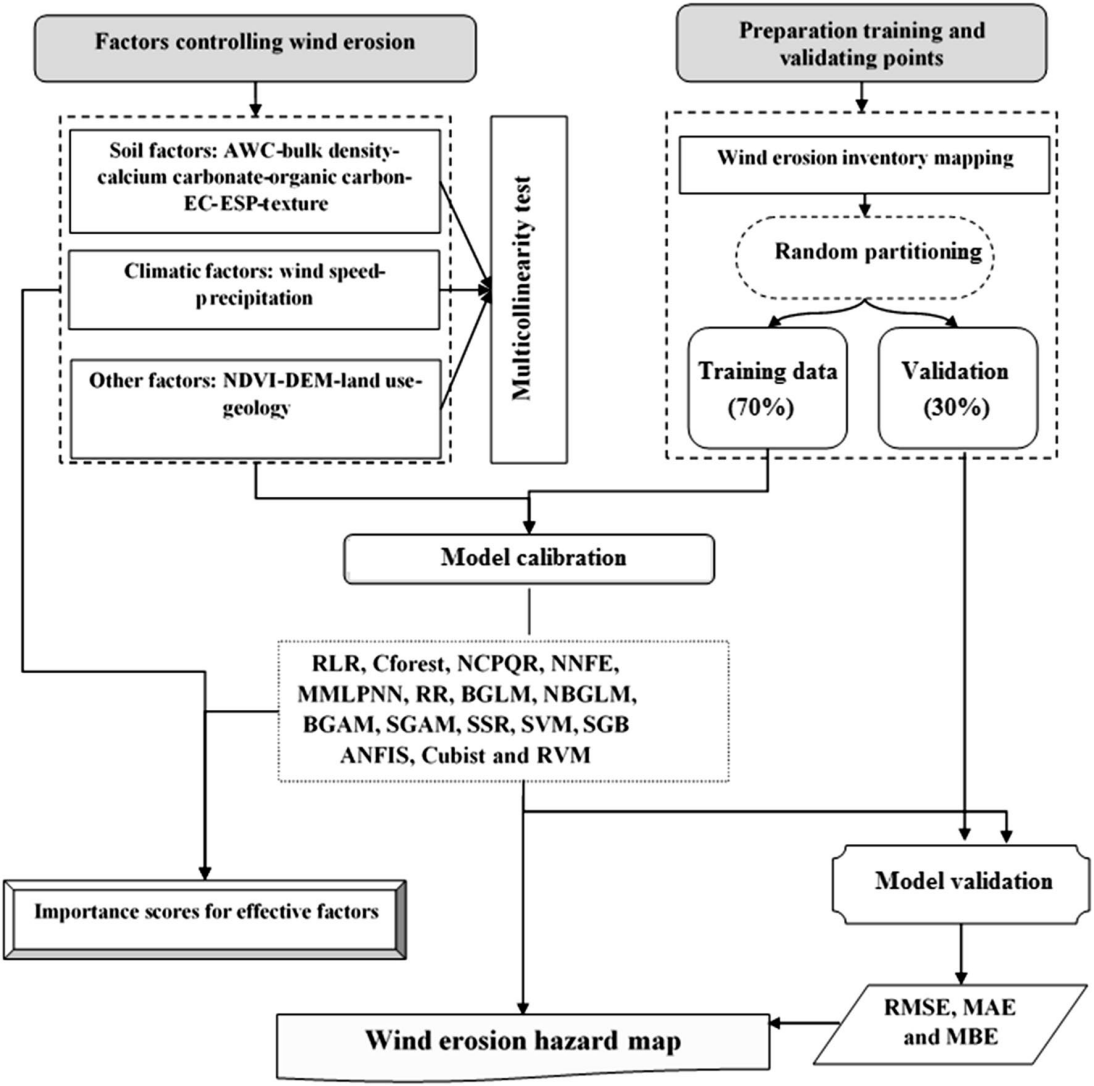
Flowchart of the methodology for mapping of wind erosion hazard.

A brief overview of the main steps used in our methods is presented in Fig. 11.
